# Gas–Solid Phase Separation of Active Brownian Particles Under Confinement of Hard Walls

**DOI:** 10.3390/nano15221746

**Published:** 2025-11-20

**Authors:** Hao Zhang, Shenghua Xu, Shuangyang Zou, Hongwei Zhou, Wenze Ouyang, Jun Zhong

**Affiliations:** 1 Key Laboratory of Microgravity, Institute of Mechanics, Chinese Academy of Sciences, Beijing 100190, China; 2 School of Materials Engineering, North China Institute of Aerospace Engineering, Langfang 065000, China; 3 School of Engineering Science, University of Chinese Academy of Sciences, Beijing 100049, China

**Keywords:** active Brownian particles, motility-induced phase separation, hard walls, gas–solid phase coexistence

## Abstract

By means of computer simulations, we have investigated the gas–solid phase separation of active Brownian particles (ABPs) under the confinement of two hard walls, distinct from the gas–liquid phase separation typically seen in bulk systems. Our results show that the distance (D) between the hard walls plays a crucial role. Increasing D may facilitate the formation of gas–solid phase separation perpendicular to the hard walls, while decreasing D may suppress such phase separation. Interestingly, when D is decreased further and the lateral system size is increased accordingly to maintain a constant volume, a new reoriented phase separation pattern in the system emerges, i.e., the gas–solid phase coexistence can be found in those layers parallel to the inner surfaces of two hard walls. These intriguing findings illustrate how ABPs can achieve simultaneous localization and crystallization under imposed boundary confinement, thereby fundamentally altering the pathway of phase separation. Also, such understanding may provide a valuable pathway for optimizing the design of systems full of active matters such as micro-robotics or targeted delivery platforms.

## 1. Introduction

Active matters may constitute a typical class of non-equilibrium systems where individuals often exhibit self-propulsion motions by means of inputting energies continuously from their surroundings [1]. Such classification has spanned systems with a wide range of performances and diverse characteristics, e.g., macroscopic animal collectives like starling groups demonstrating emergent vortex patterns [2,3], mesoscopic bacterial colonies [4], and red blood cells exhibiting intricate tumbling motions in capillary networks [5], and microscopic motor proteins like dyneins orchestrating cilia concordant beatings [6], and actin filaments generating contractile forces in muscle tissues through their interactions with myosin, etc. [7]. While these propulsion mechanisms vary substantially when crossing certain fields, e.g., from metabolic energy conversion in biological systems to self-propulsion-induced catalysis in synthetic colloids, they exhibit universal hallmarks of collective phenomena such as dynamic self-regulation, clustering, and motility-induced phase separation (MIPS), etc., which may possess characteristics of self-assembly somehow.

The active colloids, typically comprising particles with sizes ranging from nanometers to micrometers, have provided particularly tractable platforms in experiments for probing questions in non-equilibrium physics [8]. For example, size characteristics of active colloids may enable real-time optical tracking while maintaining relevance to biological processes such as leukocytes squeezing out through gaps among endothelial cells during inflammatory responses [9] and therapeutic nanorobots delivering drugs through tortuous tumor vasculature [10], both of which represent natural prototypes of confined active matter.

The ability to synthesize active colloids such as Janus particles possessing asymmetric catalytic coatings [11], or magnetically steerable micro-swimmers [12], etc., has become greatly amazing for engineers. Specifically, it may provide precise laboratory analogs for those biological scenarios. Moreover, it may provide tunable parameters such as the catalytic activity of coatings on Janus particles or the magnetic responsiveness of micro-swimmers, which are inaccessible in the live systems.

The active Brownian particle (ABP), a simple model for active colloids, has laid itself as an essential cornerstone for theoretical investigations. This could be attributed to its formulation with concise yet physical illustrations. By incorporating self-propulsion along an orientating vector subject to rotational diffusion and short-range steric repulsion, ABPs may capture essential features like MIPS emergence without requiring explicit alignments [13]. Recent experimental validations for active colloid suspensions by using Quincke rollers [14] have confirmed such a model’s wonderful power for bulk phase behaviors.

However, there still exists a significant gap between these idealized models and actual applications in the real world, which is evident in various scenarios. For instance, in the soil, the activities of active colloidal substances like microorganisms and organic particles are constrained by the pore geometries of soil [15,16]; the motions of thrombus in blood vessels are restricted by vessel diameters and the rheological properties of blood [17]; and self-propelling nanorobots can hardly operate within microtubules to deliver cargoes along cytoskeletal tracks [6]. As a generic model for micro-swimmers, ABPs have been extensively explored through numerical simulations and experiments. These investigations typically center on two- and three-dimensional free space and seldom consider the consistency with the real-world environments of micro-swimmers. However, recent studies have revealed that geometric constraints have a profound influence on the behaviors of active matter [18,19,20,21,22,23,24,25]. It is also noteworthy that even in equilibrium systems, geometric confinement can induce unexpected phase behaviors. Early studies demonstrated a solid–solid phase transition in hard disks confined between parallel walls, while subsequent work rigorously proved transverse pressure instability in confined hard disk systems [26,27]. This would naturally raise a fundamental question: How do confinement-induced geometrical constraints and boundary effects reshape collective dynamics and phase behavior of ABPs?

In this article, we implemented computer simulations of over-damped Langevin dynamics (ODLD) to observe dynamic phase behaviors of ABPs under the confinement of two hard walls. Instead of the gas–liquid phase separation previously observed in the bulk ABP system, we observed a gas–solid phase separation under the confinement of two hard walls. By varying the distance between the two hard walls, we comprehensively studied the impacts of such confined boundaries on ABPs’ gas–solid phase separation. We believe that our research may shed new light on mechanisms of phase separation in micro-swimmers under aforesaid confinement, and may provide valuable guidance for controlling micro-swimmers in practical work.

## 2. Materials and Methods

### 2.1. ABP Model

In the ABP system [28,29,30,31], spherical particles with a diameter of *σ* may have interactions via a purely repulsive Weeks–Chandler–Anderson (WCA) potential

(1) $$U\left(r_{ij}\right)=\left\{\begin{array}{ll} 4\varepsilon_{w}\left[{\left(\frac{\sigma }{r_{ij}}\right)}^{12}-{\left(\frac{\sigma }{r_{ij}}\right)}^{6}+\frac{1}{4}\right], & r_{ij}\leq \;\sqrt[{6}]{2}\sigma \\ 0, & r_{ij}>\sqrt[{6}]{2}\sigma \end{array}\right.,$$

where *r_ij_* was the center distance between particle *i* and particle *j*, *ε_w_* was the energy well of the WCA potential. The translational motion of ABP was described by the over-damped Langevin equation (the so-called ODLD)

(2) $$\dot{r}\left(t\right)=\frac{D_{T}}{k_{B}T}F\left(t\right)+v_{0}e+\Gamma \left(t\right),$$

where ***r*** was a particle’s vector position, *v*_0_ was the self-propulsion speed of a particle, ***D_T_*** was the translational diffusion coefficient, *k_B_* was the Boltzmann constant and *T* was the surrounding temperature. And, F=−∇U was the resultant force exerting on the ABPs. The Gaussian and Markovian random force, which represented thermal fluctuations, **Γ**(t), obeyed a zero mean value under well-defined second-order moments, i.e.,

(3) $$\left\langle \Gamma_{\alpha }\left(t\right)\Gamma_{\beta }\left(t^{\prime }\right)\right\rangle =2D_{T}\delta_{\alpha \beta }\left(t-t^{\prime }\right),$$

where *α* and *β* denoted Cartesian components.

The orienting vector ***e*** changed independently along with the following equation

(4) $$\dot{e}\left(t\right)=e\left(t\right)\times \hat{\eta }\left(t\right).$$

Here, $\hat{\eta }\left(t\right)$ was related to the rotational diffusion coefficient *D_R_*, which was a random variable obeying

(5) $$\left\langle \hat{\eta }\left(t\right)\cdot \hat{\eta }\left(t^{\prime }\right)\right\rangle =2D_{R}\delta \left(t-t^{\prime }\right).$$

In the low-Reynolds-number regime, the relationship between translational and rotational diffusion coefficients in 3D was $\frac{\sigma^{2}D_{R}}{D_{T}}=3$.

### 2.2. Péclet Number

To quantify the competition between self-propulsion motion and diffusive transport among ABPs, an important dimensionless parameter named the Péclet number (*P_e_*) was defined as follows:

(6) $$P_{e}=\frac{v_{0}}{\sigma D_{R}}.$$

Following Ref. [28], we fixed the ratio of self-propulsion force to thermal energy, which could consistently maintain the collision dynamics among particles. In particular, it ensured a constant effective diameter during oblique collisions. Specifically, we set the relationship as

(7) $$\frac{k_{B}Tv_{0}\sigma }{\varepsilon_{w}}=24.$$

This constraint also ensured that the range of repulsive interactions remained invariant throughout the whole ODLD simulations.

### 2.3. Identification of Crystallization

**Figure 1:**
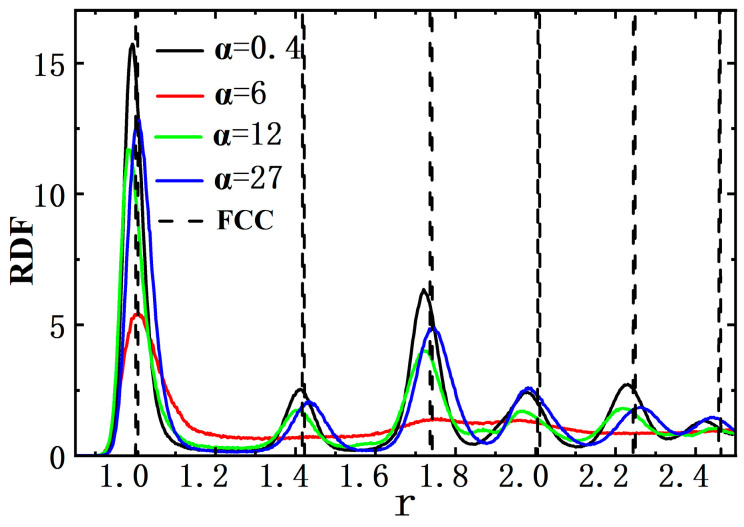
Radial distribution function (RDF) for active Brownian particle (ABP) system. The horizontal type axis was the radial distance *r*, while the vertical axis was the RDF values. The dashed line showed the RDF distribution for a perfect FCC lattice under density *ρ* = 1.4 (*ϕ* = 0.733).

In order to distinguish crystal particles from liquid ones, we adopted an approach offered by Frenkel and coworkers [32,33], with which bond-orientated order parameters were calculated by means of spatial arrangements of neighbor particles. That was, for a particle *i*, we first defined all the particles within a given radius *r*_c_ as its nearest neighbors. Note that here *r*_c_ = 1.2*σ* was selected to be near the first minimum of the radial distribution function (RDF) in the ABP system (see Figure 1). As a result, the parameter of complex bond-oriented order $q_{lm}(i)$ was calculated as follows [34]:

(8) $$q_{lm}\left(i\right)=\frac{1}{N_{nb}\left(i\right)}\sum_{j=1}^{N_{nb}\left(i\right)}Y_{lm}\left(r_{ij}\right),$$

where $N_{nb}(i)$ was the number of nearest neighbors for particle *i*, $Y_{lm}(r_{ij})$ was a spherical harmonic function, *l* was a free integer parameter (angular momentum), and *m* was an integer ranging from −*l* to *l*. Based on Equation (8), the normalized complex vector d_6m_(*i*) was computed as follows:

(9) $$d_{6m}\left(i\right)=\frac{q_{6m}\left(i\right)}{{\left[\sum_{m=-6}^{6}{\left|q_{6m}\left(i\right)\right|}^{2}\right]}^{1/2}}.$$

Then the structural correlation between two neighboring particles *i* and *j* was quantified by the following scalar product:

(10) $$S_{ij}=\sum_{m=-6}^{6}d_{6m}\left(i\right)\cdot d_{6m}^{*}\left(j\right),$$

where $d_{6m}^{*}(j)$ denoted the complex conjugate of $d_{6m}^{*}(i)$. Here, two neighboring particles *i* and *j* were considered as being connected if $S_{ij}\geq 0.70$. And, if a particle had at least seven such connections with its neighboring particles, it could be classified as a solid object. Otherwise, it was still labeled as a liquid type.

### 2.4. Numerical Simulations

In computer simulations, the reduced units were used [35], e.g., *σ*, *ε_w_* and *τ_R_* = 1/*D_R_* (rotational diffusion time) were selected as basic scales for length, energy, and time, respectively. The equations of ABPs’ motion (Equations (2) and (4)) were solved numerically with a time step *δt* = 4.0 × 10^−5^. Specifically, the translational equation (Equation (2)) was integrated using the Ermak–McCammon algorithm, while the orientational equation (Equation (4)) was solved by integrating stochastic angular increments in spherical coordinates followed by vector renormalization, as described in Ref. [30].

In this work, generally, we aimed at studying kinetics of ABPs’ phase behaviors under the confinement of two hard walls. The ODLD simulations were carried out in an *NVT* ensemble, i.e., the number of ABPs (*N*), the volume of the ABP system (*V*) and the surrounding temperature (*T*) exerting on the system were kept constant. Also, along the *Z*-direction, those two hard walls parallel to the *XY*-plane were put at the top and bottom sides of the box, respectively. According to these, the simulation box had periodic boundaries along the *X*- and *Y*-directions but a fixed boundary along the *Z*-direction. When any of the ABPs attempted to penetrate into the two hard walls, it would be bounced back into the simulation box along the *Z*-axis. In addition, the ABP system was characterized by an aspect ratio α = L_x_/L_z_ (L_y_/L_z_), where L_x_ (L_y_) and L_z_ were box lengths along the *X*(*Y*)- and *Z*-directions, respectively (here we kept L_x_ = L_y_). As a result, if *α* ≤ 1.0, the box elongated along the *Z*-axis, the distance between two hard walls became larger and larger, while the box lengths along the *X*- and *Y*-axes shrank accordingly. Similarly, if *α* > 1.0, the distance between two hard walls decreased while box lengths along the *X*- and *Y*-axes increased. Apparently, the above operations obeyed a rule: The volume of the ABP system must be kept constant (of course the density of ABP must remain constant) during all the ODLD simulations under the *NVT* ensemble. Therefore, *α* could be regarded as a key factor to induce phase separation in the computer simulations.

During the ODLD simulations here, the ABP system was composed of 131,072 particles initially laid out in a 3D cubic box. To make the computer simulations run faster and more efficiently, the graphics processing unit (GPU)-accelerated parallel algorithm was selected to be implemented on a platform of Compute Unified Device Architecture (CUDA) [36]. Before running the simulations, we randomly put those ABPs into the simulation box, ensuring that there were no overlapping positions among them. Here, the number density of ABPs: *ρ* = *N*/*V* = 0.8 (liquid type), the local packing fraction*: ϕ =* π*ρ*/6*,* and the Péclet number: *P_e_* = 100. So under the conditions above, the phase separation could be successfully observed during all the simulations [13].

## 3. Results and Discussion

### 3.1. Gas–Solid Phase Separation

As is known, no gas–liquid phase separation can occur in WCA fluid, a typical equilibrium system, due to the absence of attractive interactions. However, the system under investigation in the present study is composed of ABPs, which have self-propulsion motion. In such a non-equilibrium system, the self-propulsion of the particles gives rise to an effective “attraction” that can lead to phase separation, a phenomenon known as MIPS. Previously, the bulk ABP system was reported to often have a gas–liquid phase separation under high enough *P_e_* [28,37,38,39,40,41], although the WCA interaction between ABPs is purely repulsive (see Equation (1)).

**Figure 2:**
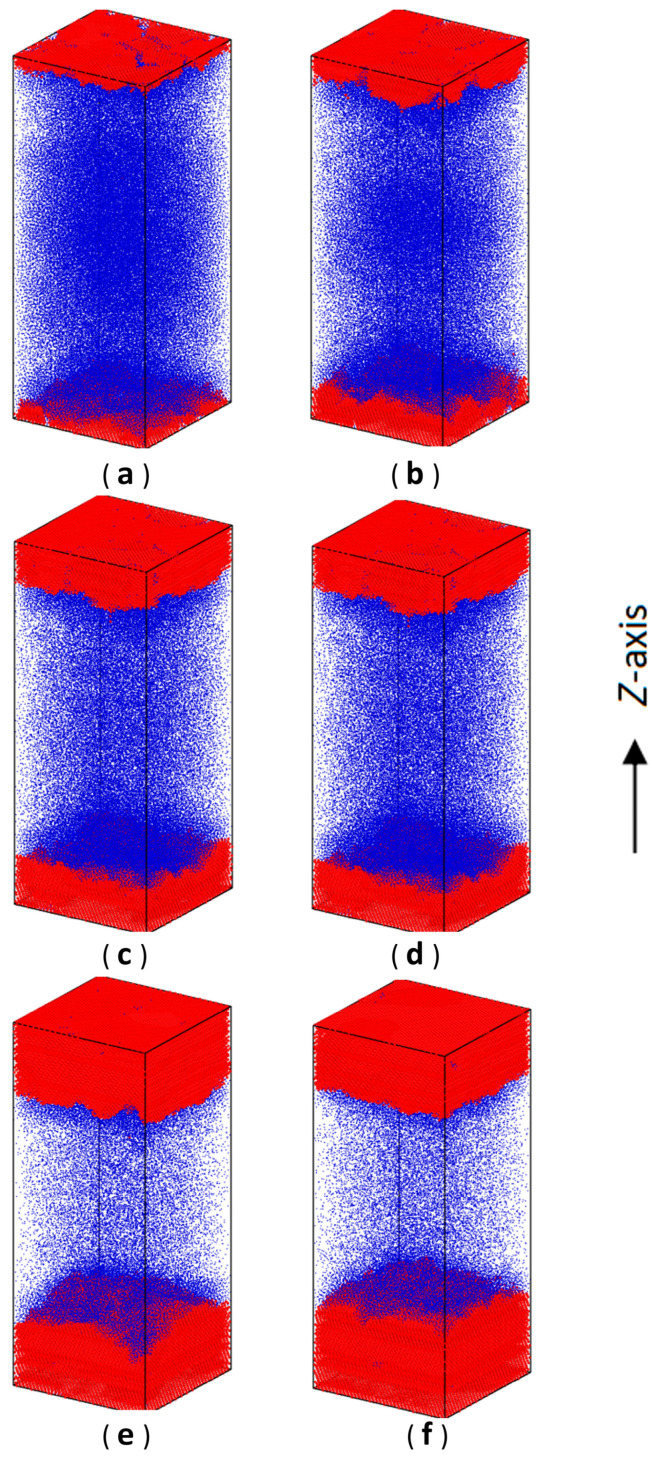
Snapshots (visualized by the OVITO software) to illustrate processes of motility-induced phase separation and crystallization at *α* = 0.4, *P_e_* = 100. In the color scheme: blue spheres represented disordered particles; red ones denoted crystalline particles. (**a**) *t* = 1 × 10^5^·*δt*; (**b**) *t* = 2 × 10^5^·*δt;* (**c**) *t* = 3 × 10^5^·*δt*; (**d**) *t* = 4 × 10^5^·*δt*; (**e**) *t* = 1 × 10^6^·*δt*; (**f**) *t* = 5 × 10^6^·*δt*.

During the numerical simulations here, we initially set up a large space between two hard walls at *α* = 0.4 (L_z_ = 100.8) and put ABPs into this vacuum system (a liquid type). Next, the phase evolution (separation) in the system was observed. In contrast to the gas–liquid phase separation observed in the previous literature [37,41], at this time, we clearly observed one disordered and two ordered domains to generate in the ABP system (the gas–solid phase separations). As shown in Figure 2 and Supplementary Video S1, some typical snapshots using the Ovito 3.11.0 software [42] were selected for verifying such phase evolution (α = 0.4). Specifically, we used the bond orientational order parameters to distinguish solid particles from liquid ones, as described in Section 2.3. Obviously, at first, the ABP crystallization (two ordered domains) started near the inner surface regions of two hard walls. Then, along the *Z*-axis (the direction perpendicular to inner surfaces of two hard walls), the crystallization began to extend towards the middle region of the simulation box. From this point of view, such ABP crystallization was similar to the colloidal epitaxy with the induction of templates or hard walls [43,44,45,46].

**Figure 3:**
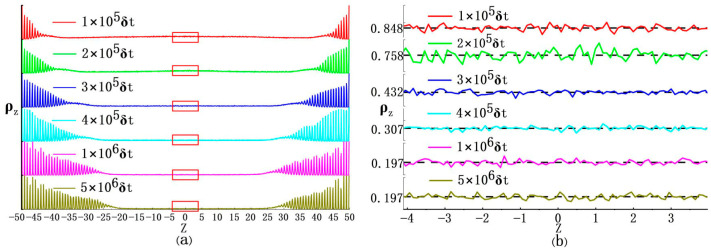
Density profiles along the *Z*-axis corresponding to snapshots in Figure 2. (**a**): The density profiles were calculated by dividing the simulation box length along the *Z*-axis into 1000 bins. The curves were presented in ascending order of simulation time (as indicated in the panels), from top to bottom. (**b**): Enlarged views of regions marked by red boxes in (**a**).

In detail, during the ODLD simulations here, we collected density profiles of ABPs along the *Z*-axis under different time steps. As shown in Figure 3, many sharp peaks on density curves showed crystalline features near the inner surface regions of two hard walls (two ordered domains), while far from two hard walls, these curves became much smoother to indicate disordered features (one disordered domain). Also, density values in the disordered domain were much smaller than those in the two ordered ones (*ρ* ≈ 1.4 and *ϕ* = 0.733) [13]. Finally, the ABP system reached a stable state, i.e., the sections on density curves representing ordered domains stopped extending towards the middle region of the simulation box and showed small fluctuations throughout the whole *Z*-axis. Please note that the final density values in the disordered domain at the middle of the simulation box were approximately 0.197, which was very close to the gas phase obtained from a phase diagram for a 3D ABP system [13]. Based on this, we may conclude that such phase separation induced by two hard walls should be regarded as the gas–solid phase separation, not the liquid–solid feature.

**Figure 4:**
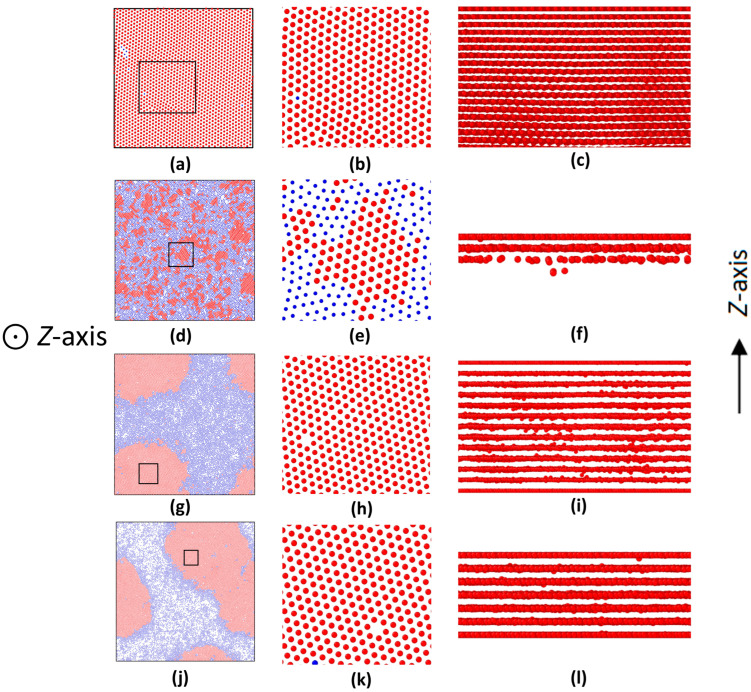
Configurations near two hard walls at different aspect ratios (*α* = L_x_/L_z_) at *P_e_* = 100. Particles were colored blue (disordered) or red (crystalline). (**b**,**e**,**h**,**k**): Enlargements of black-framed areas in (**a**,**d**,**g**,**j**), respectively, revealing a hexagonal-type lattice structure of crystals. (**c**,**f**,**i**,**l**): Crystalline layers along the *Z*-axis for (**a**,**d**,**g**,**j**), respectively. (**c**,**f**) showed the portion of crystal layers, while (**i**,**l**) showed complete layers. (**a**) *α* = 0.4, *t* = 5 × 10^6^·*δt*; (**d**) *α* = 6, *t* = 2 × 10^7^·*δt*; (**g**) *α* = 12, *t* = 5 × 10^6^·*δt*; (**j**) *α* = 27, *t* = 5 × 10^6^·*δt.*

For the two ordered (crystalline) domains obtained above, it seemed that the multi-layered textures in them were hexagonal (see Figure 4a,b), i.e., their nearest-neighbor distances (similar to lattice constants) were close to 1.0 which matched the (111)-plane in a face-centered cubic (FCC) crystal type for *ρ* ≈ 1.4 (*ϕ* = 0.733). Thus, there was no doubt that such crystalline textures induced by two hard walls corresponded to the (111)-plane in an FCC crystal, as had been observed in the hard-wall-induced crystallization of passive colloids previously [47]. To further analyze more detailed information about the ABP system, we also applied the radial distribution function (RDF) to the final configurations in the ABP system (see Figure 1). For the validation of structural assignments, we compared the RDF curves of the ABP system with those of an ideal FCC crystal type. The result indicated that, despite slight deviations, those positional alignments confirmed the predominance of FCC symmetry in the two ordered domains of the ABP system (see Figure 1).

### 3.2. Inner Surface Effects on ABPs

Although the crystallization induced by two hard walls performed similarly to the colloidal epitaxy, such phase behavior in the ABP system was still quite different from those of passive colloids. In fact, when the crystallization in the ABP system took place, the MIPS also occurred simultaneously because of the ABP inherent activities (see Figure 2 and Figure 3) [23,48,49]. For passive colloids, crystals can be formed under suitable (smaller) thermodynamic constraints and large enough density values of particles. According to these, for the ABP system, (1) two hard walls here could be thought to induce the crystallization by reducing free-energy barriers in the ABP system [50]. (2) Since the crystals were typically formed under non-equilibrium states by means of thermal interactions and self-driven effects on active particles [23,48,49], the inner surfaces of two hard walls here, which were proved to have an “attractive” effect on self-propelling particles [21,23,47,51,52,53,54], may cause ABPs to move towards them, so that ABPs may accumulate higher and higher densities near the regions of the two hard walls. Therefore, when the density of the ABPs near these regions reached a critical point, the crystallization (crystal nucleation) would take place. And then, they would extend towards the middle region of the simulation box.

Furthermore, we assumed that the preferential formation of crystals was likely dependent upon the non-equilibrium crystallization process correlated with the classical “nucleation-growth” framework. Similarly, in the ABP system, high density values of ABPs in regions near two hard walls may provide preferred nucleation sites, i.e., when nucleation sites in these regions appeared, crystalline layers could be generated. Next, these layers extended along the *X*- and *Y*-axes and become thicker along the *Z*-axis, accompanied by crystallization and gas–solid phase separation in the ABP system (see Figure 2 and Figure 3). The picture described above was reminiscent of the epitaxial growth on crystalline fronts, which has been widely applied to manufacturing the thin-film materials [55]. Here the process of layer growth looked like the Frank-van der Merwe (FM) mode in epitaxy. However, configurations on each layer front looked pretty rough, not like the typical FM mode with ideal 2D planes (see Figure 2). This may be due to thermal fluctuations in the computer simulations. Moreover, when such crystallization plus gas–solid phase separation reached a stable state, two formed crystalline domains hardly moved as a whole, not like other ones formed by active colloids [56,57]. This was possibly due to interactions of “multibody caging effects” [13,30] between two hard walls and crystalline domains [19,58].

Readers may inquire why results in this work do not show the gas–liquid phase separation as reported in the previous literature [28,37]. It was mainly due to the interactions between two hard walls and the ABPs [23,51]. In detail, a previous phase diagram for the 3D ABP system [28,37] indicated that the gas–liquid binodal was enclosed within the gas–solid coexisting region. Here if the ABP system was under three periodic boundaries along *X*-, *Y*-, and *Z*-axes, it would result in MIPS and stay at a metastable state full of gas–liquid coexisting phases for an extensively long time [48]. However, if exerting two hard walls on this system, the ABPs seemed to form dynamic clusters near two hard walls mainly due to their self-propelling forces. But in fact, two hard walls would induce the ABPs accumulation more likely than their spontaneous collection. According to these, at an initial stage of crystallization (see right panels in Figure 3) in the ABP system, there would also appear several dense ABP clusters far from two hard walls, in which their ABP densities were larger than their initial ones (*ρ* = 0.8 and *ϕ* = 0.419)—the so-called gas–liquid coexistence. But in the next steps, competitions of phase separation between the spontaneous gas–liquid phase and the hard-wall-induced gas–solid phase would take place. Ultimately, the latter overwhelmed with the former to dominate the whole ABP system and facilitated itself to be the steady state in the system.

### 3.3. Confinement of Hard Walls

**Figure 5:**
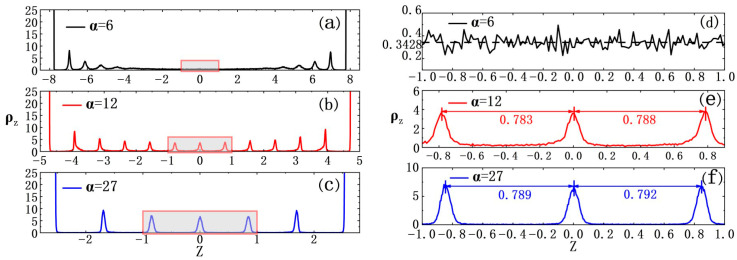
Density profiles along the *Z*-axis for different aspect ratios (*α*) at *P_e_* = 100. The simulation times are: (**a**) *t* = 2 × 10^7^·*δt*; (**b**,**c**) *t* = 5 × 10^6^*δt.* (**d**,**e**,**f**): Enlargements of regions marked by red boxes in (**a**–**c**), respectively. The black dotted line in (**d**) indicated an average density value within selected regions. Solid arrows in (**e**,**f**) indicated the distance between adjacent peaks. In a perfect FCC (111) crystal with density *ρ* = 1.4 (*ϕ* = 0.733), the facet space of 0.817 lied within 5% of measured peak distance as shown in the figures.

Performance of gas–solid phase separation in the ABP system would vary with the distance between two hard walls. For example, when the distance between two hard walls was decreased to *α* = 6.0 (L_z_ = 16.6), although ABPs still formed crystals in the space between two hard walls, the crystals were quite different from those at smaller *α* values. That was, such crystallization did not finally form two distinct solid domains near two hard walls. Instead, it evolved into lots of small crystalline clusters (see Figure 4d and Supplementary Video S2). As shown in Figure 5a, during the ODLD simulations, density profiles along the *Z*-axis indicated so that initially a few small crystalline clusters appeared near two hard walls (see also Figure 4f). As the simulation progressed, such density profiles became significantly larger than those of the gaseous phase in the middle region of the simulation box (see Figure 5d). This meant that, under current confinement of two hard walls, a sole gas–solid phase separation did not take place. Next, the ODLD simulations were conducted for a much longer time (*t* = 10^8^·*δt*), and we still did not see the completion of gas–solid phase separation and the merge of small crystalline clusters.

If we continuously decreased the distance between two hard walls, for example, at *α* = 12 (L_z_ = 10.4, and the box lengths along the *X*- and *Y*-axes were extended more to meet this large *α*; see Supplementary Video S3), along the *Z*-axis, the two hard walls had strong effects on ABP motions because their “interaction” ranges began to overlap, and they could be linked by the crystalline layers (see Figure 4i and Figure 5b).

In principle, to generate the typical coexistence of the gas–solid phase, it did need a space big enough to hold such phase textures. However, with the decrease in the space between two hard walls here, there was no doubt that the gas–solid phase separation along the *Z*-axis was thoroughly suppressed till it totally vanished. Instead, the ABPs finally evolved into constructing more layers (the *XY*-layers) full of crystalline clusters. From this point of view, the two hard walls here should play an annihilating role in the gas–solid phase separation. Please note: with the decrease in the space between two hard walls, box lengths along *X*- and *Y*-directions should be extended so that the whole box volume could still remain constant .

**Figure 6:**
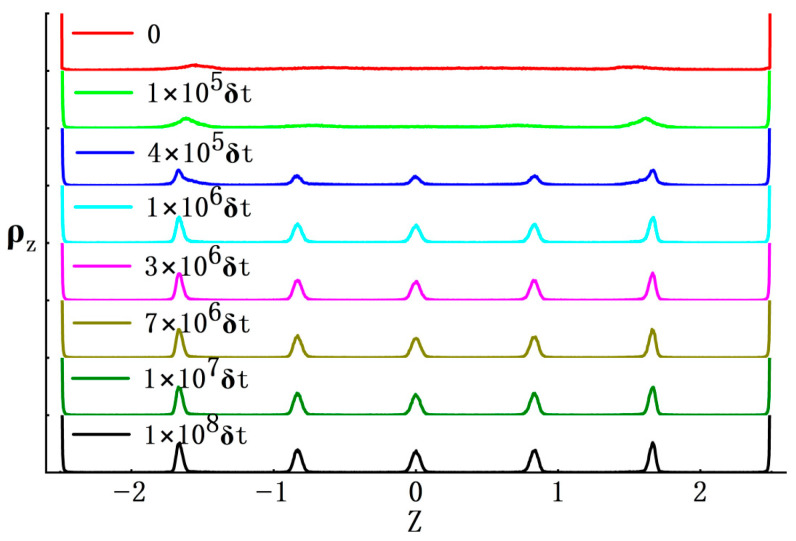
Density profiles along the *Z*-axis corresponding to the evolution of the ABP system shown in Figure 7. The density profiles were calculated by dividing the simulation box length along the *Z*-axis into 1000 bins. The curves were presented in the order of increasing time (as indicated in the panels), from top to bottom.

**Figure 7:**
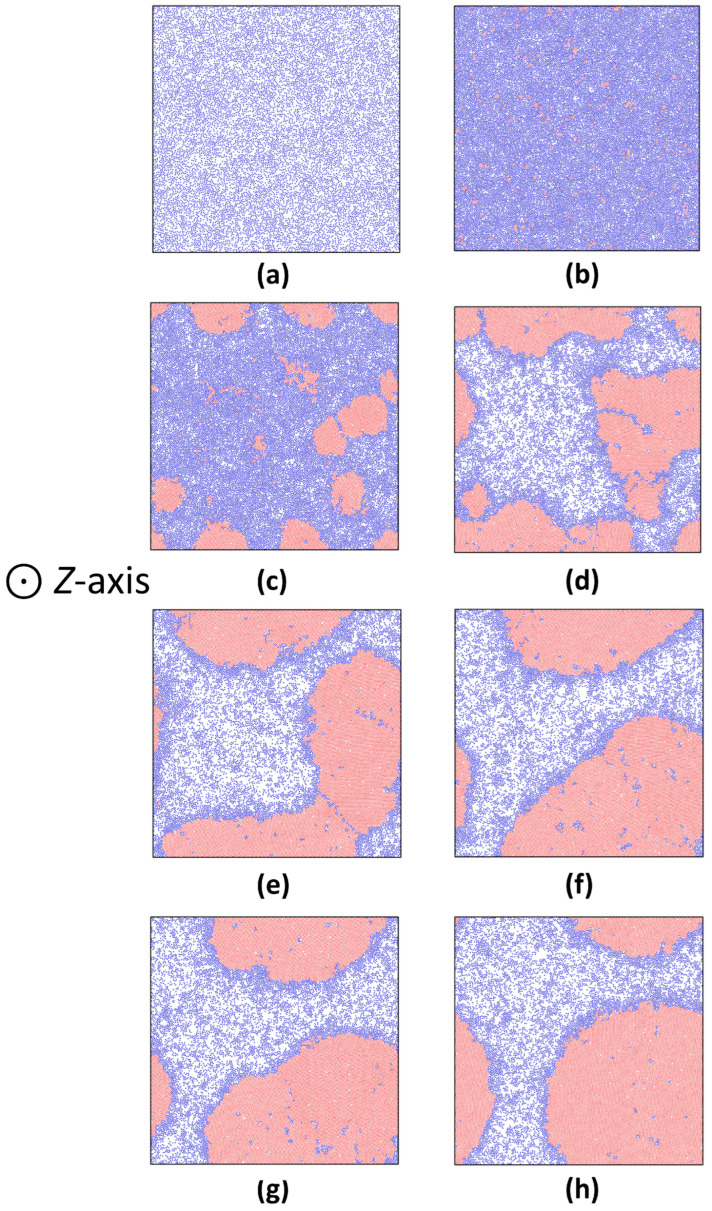
Evolution of ABPs near two hard walls at *α* = 27, *P_e_* = 100. (**a**) *t* = 0 (initial configuration); (**b**) *t* = 1 × 10^5^·*δt* (particles adhered to hard walls); (**c**) *t* = 4 × 10^5^·*δt* (onset of phase evolution); (**d**) *t* = 1 × 10^6^·*δt* (ongoing phase evolution); (**e**) *t* = 3 × 10^6^·*δt* (completed phase evolution); (**f**) *t* = 7 × 10^6^·*δt* (crystals began to drift); (**g**) *t* = 1 × 10^7^·*δt* (crystals continued to drift); (**h**) *t* = 1 × 10^8^·*δt* (crystal shape became circular).

To further analyze the mechanisms of gas–solid phase evolution in the ABP system under extreme confinement of two hard walls, here we even set *α* = 27 (L_z_ = 6.1; see Supplementary Video S4), at which the ABP system was compressed along the *Z*-axis so tightly that the distance between the two hard walls was merely about a few particle diameters (see Figure 6). This meant that the two hard walls had much stronger impacts on ABP motions. In addition, box lengths along *X*- and *Y*-axes must be extended much farther than those at *α* = 12. To obtain more valuable information, here we ran the computer simulations under a much longer time (*t* = 10^8^·*δt*). The results indicated that initially the ABP clusters in formed layers (the *XY*-layers) were found to distribute uniformly (Figure 7a,b). Next, when the simulation time went on, crystalline clusters in each of the layers began to evolve transversely: they looked like “islands” drifting along the *X*- and *Y*-axes, and merging into bigger crystalline ones (see Figure 7, the so-called cluster coalescence). Finally, a new phase type of gas–solid coexistence was found to form in those layers with diverse configurations, as shown in Figure 7c–h). The reason could be explained as follows: In the current situation, box lengths of the ABP system along *X*- and *Y*-axes were extended so much farther that they could free up a huge transverse space to hold new gas–solid coexisting textures. This was called the reoriented phase separation, which may also resemble the Volmer–Weber mode in epitaxy [55].

Another interesting discovery was that those big crystalline clusters may rearrange their geometries during the coalescence: At the early stage (Figure 7e) when they began forming, their outlines seemed to be irregular. But after a long-time evolution, they would become more and more circular in the *XY*-layers (Figure 7h). This self-healing mechanism, absent in traditional equilibrium crystal coalescence, was a hallmark of active systems, and resembled the defect-resilient behaviors of bacterial biofilms [59,60].

## 4. Conclusions

As is well known, the bulk ABP system usually tends to form a gas–liquid phase separation at high activity (the Péclet number *P_e_* is very large). In this work, we revealed distinct non-equilibrium phase behaviors in the 3D ABP system under the confinement of two hard walls. If ABPs with a high enough *P_e_* were put into a space confined by two hard walls, the crystallization or freezing near the two hard walls occurred, and the distinct gas–solid phase separation preferably took place instead of the conventional gas–liquid phase separation. In this situation, the distance between the two hard walls significantly influenced the characteristics of gas–solid phase separation, i.e., when the distance between the two hard walls became large enough, the gas–solid phase separation distinctly formed along the *Z*-axis. However, when the two hard walls were compressed to shorten their distance, this gas–solid phase separation was further suppressed along the *Z*-axis, until it totally vanished. If we further decreased the distance between the two hard walls and simultaneously extended the box lengths parallel to the hard walls to maintain the whole system volume constant, the gas–solid phase separation was observed to evolve into the new phase type of gas–solid coexistence in layers parallel to the hard walls.

Our findings underscore a dual role of hard-wall boundaries on the ABP system: the confined hard walls facilitate crystalline accumulations of active particles, but meanwhile they can also influence the characteristics of phase separation. These insights offer valuable and practical guidelines for optimizing the design of the ABP system under extreme confinement from its surroundings. For instance, in applications of programmable drug delivery vehicles, some tiny “robots” in confined micro-/nano-space, and some biomarkers for disease diagnosis in microfluidic chips, the ability to control their phase behaviors should be of crucial significance.

Although our present work may provide some valuable clues for the behaviors of active colloids under 3D environmental confinement, it does need experimental testing to verify such behaviors. Even if actual situations in the real world are more complicated than those expected here, it would be interesting to extend the present framework to systems with polydisperse or chiral active particles [6,31,61,62,63], where several collective phase behaviors are still unknown. Of course, in the real world, confinement on the system from its surroundings would not be consistent with the ideal case of two hard walls here. However, in principle, properties of hard walls like their shape, structure and interactions with those active particles should reveal many unknown phase behaviors in the active system.

## Data Availability

The original contributions presented in this study are included in the article/Supplementary Material.

## Supplementary Materials

The following supporting information can be downloaded at: https://www.mdpi.com/article/10.3390/nano15221746/s1, Supplementary Video S1: Evolution of Configuration at *α* = 0.4, Supplementary Video S2: Evolution of Configuration S2 at *α* = 6, Supplementary Video S3: Evolution of Configuration at *α* = 12, Supplementary Video S4: Evolution of Configuration at *α* = 27.

## Abbreviations

The following abbreviations are used in this manuscript:

ABP	Active Brownian Particle
MIPS	motility-induced phase separation
ODLD	over-damped Langevin dynamics
WCA	Weeks-Chandler-Anderson
RDF	radial distribution function
GPU	Graphics Processing Unit
CUDA	Compute Unified Device Architecture
FCC	face-centered cubic
FM	Frank-van der Merwe
